# Ubiquitin modifications

**DOI:** 10.1038/cr.2016.39

**Published:** 2016-03-25

**Authors:** Kirby N Swatek, David Komander

**Affiliations:** 1 Medical Research Council Laboratory of Molecular Biology, Francis Crick Avenue, Cambridge, CB2 0QH, UK

**Keywords:** ubiquitin, proteasomal degradation, phosphorylation, post-translational modification, Parkin

## Abstract

Protein ubiquitination is a dynamic multifaceted post-translational modification involved in nearly all aspects of eukaryotic biology. Once attached to a substrate, the 76-amino acid protein ubiquitin is subjected to further modifications, creating a multitude of distinct signals with distinct cellular outcomes, referred to as the 'ubiquitin code'. Ubiquitin can be ubiquitinated on seven lysine (Lys) residues or on the N-terminus, leading to polyubiquitin chains that can encompass complex topologies. Alternatively or in addition, ubiquitin Lys residues can be modified by ubiquitin-like molecules (such as SUMO or NEDD8). Finally, ubiquitin can also be acetylated on Lys, or phosphorylated on Ser, Thr or Tyr residues, and each modification has the potential to dramatically alter the signaling outcome. While the number of distinctly modified ubiquitin species in cells is mind-boggling, much progress has been made to characterize the roles of distinct ubiquitin modifications, and many enzymes and receptors have been identified that create, recognize or remove these ubiquitin modifications. We here provide an overview of the various ubiquitin modifications present in cells, and highlight recent progress on ubiquitin chain biology. We then discuss the recent findings in the field of ubiquitin acetylation and phosphorylation, with a focus on Ser65-phosphorylation and its role in mitophagy and Parkin activation.

## Introduction

The realization by Goldknopf *et al*. that histones can be modified by the protein ubiquitin through Lys-linked isopeptide bonds^1^ marked a new era in post-translational signaling. At the time, small chemical modifications of amino acids, including phosphorylation and acetylation, were already known to regulate protein function^2,3^. The prevalence and importance of protein-based modifications emerged in the 1980s, when landmark studies connected the ATP-dependent ubiquitination of substrates to their degradation by the 26S proteasome^4^. 35 years later, it is clear that this function of ubiquitin was just the tip of an enormous iceberg.

The post-genomic era provided insight into the complexity of the ubiquitin system, and far over 1 000 proteins regulate ubiquitination in human cells^5^. Ubiquitin is attached to substrates by a sophisticated three-step enzymatic cascade^6^, utilizing E1 ubiquitin activating^7^, E2 ubiquitin conjugating^8^ and a variety of E3 ubiquitin ligating enzymes^9,10,11^. Ubiquitinated proteins are recognized by receptors that contain ubiquitin-binding domains (UBDs)^12^, and several specialized families of proteases, the deubiquitinases (DUBs), remove ubiquitin modifications^13,14^. Comprehensive proteomics studies identified tens-of-thousands of ubiquitination sites on thousands of proteins^15,16,17^. It appears that most proteins will experience ubiquitination at some point in their cellular lifetime.

Ubiquitination starts by the attachment of a single ubiquitin molecule to a substrate Lys residue. These monoubiquitination events are abundant^18^ and have many roles in cells^19^. This review focuses on the multitude of signals that can be generated when the attached monoubiquitin is modified further. We have previously referred to this complexity in the ubiquitin system as the 'ubiquitin code'^19^, borrowing nomenclature from the histone modification field. The 'histone code' describes the complex interplay of modifications that histones are subjected to, and for which many rules of interdependent, hierarchical assembly and disassembly have been functionally dissected^20^. As will be discussed below, the understanding of the ubiquitin code has advanced rapidly in recent years, however, new methods and insights are required to comprehensibly crack this complex code of post-translational modifications.

## Ubiquitin modifications ― a conceptual overview

**Figure 1:**
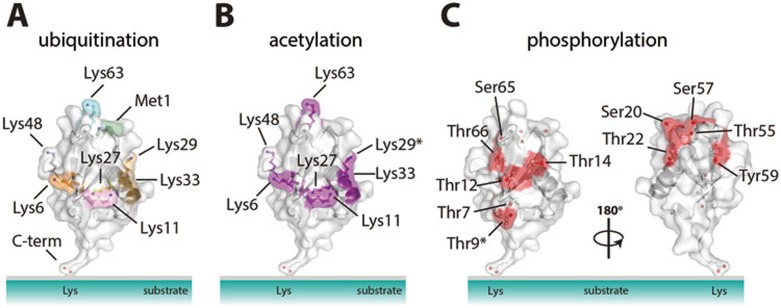
Modification sites on ubiquitin. Ubiquitin is shown as a cartoon under a semitransparent surface, and modifiable residues are shown in ball-and-stick representation with blue nitrogen and red oxygen atoms. **(A)** Structure of ubiquitin highlighting the eight sites of ubiquitination. **(B)** Six out of seven Lys residues on ubiquitin have been reported to be acetylated in proteomics datasets. An asterisk marks the seventh Lys, Lys29, for which acetylation has not been identified to date. **(C)** Identified phosphorylation sites of ubiquitin are displayed according to proteomic analysis. Red spheres indicate phosphorylatable hydroxyl groups on Ser/Thr and Tyr residues. The structure was rotated 180 degrees to show all phosphorylation sites. An asterisk on Thr9 indicates this site is ambiguously assigned.

Ubiquitin is a 76-amino acid protein, and as such bears many potential sites for additional post-translational modifications. The key features of ubiquitin are its seven Lys residues, all of which can be ubiquitinated, to give rise to isopeptide-linked ubiquitin chains. An eighth chain type, Met1-linked or 'linear' chains, is generated when ubiquitin is attached to the N-terminus of a second ubiquitin (Figure 1A). Proteomics studies show that all possible linkage types co-exist in cells^16,17,21,22,23^. Lys48-linked chains are the predominant linkage type in cells (often > 50% of all linkages, see references above), and their role is to target proteins to the proteasome for degradation^4^. In contrast, the second most abundant chain type linked via Lys63 performs various non-degradative roles^24^. More recently, research has begun to characterize the remaining, 'atypical' ubiquitin modifications (linked through Met1, Lys6, Lys11, Lys27, Lys29 or Lys33). Surprisingly, this has led to the discovery of highly linkage-specific enzymes and proteins that assemble, recognize and hydrolyze each ubiquitin chain type, i.e., 'write', 'read' and 'erase' this first layer of the ubiquitin code. On the basis of previous reviews on this topic^19,25^, the first part of this review gives an update to the exciting developments in ubiquitin chain signaling, providing a conceptual framework for future discoveries.

New layers of the ubiquitin code are currently emerging, based on findings that ubiquitin can not only be ubiquitinated, but also be modified by other modifications. These include modifications by the small ubiquitin-like (Ubl) modifier (SUMO) family^26,27,28^. SUMOylation of (poly)ubiquitin is conceptually similar to polyubiquitin chain formation. It is less clear whether other Ubl modifiers are attached to ubiquitin under physiological conditions.

More strikingly, ubiquitin is also modified by small chemically distinct post-translational modifications such as phosphorylation and acetylation. Mining of available datasets indicates that 6 out of 7 ubiquitin Lys residues can become acetylated^29,30,31,32^ (Figure 1B). Ubiquitin phosphorylation on Ser57 has been identified in some of the earliest ubiquitin proteomics experiments^15^. Subsequent studies confirmed this finding and further revealed a broad array of phosphorylation sites scattered across the surface of ubiquitin (Figure 1C). These sites include Thr7, Thr12^33^, Thr14^34^, Ser20^35^, Thr22, Thr55, Thr66^36^, Tyr59^37^, and Ser65^38^. This cornucopia of modifications on ubiquitin has a vast potential to provide additional regulation in the ubiquitin system.

Despite their existence, the kinases and acetyltransferases ('writers'), phosphatases and deacetylases ('erasers') and phospho-ubiquitin (phosphoUb) or acetyl-ubiquitin (AcUb) binding domains ('readers') for these modifications are mostly unknown, with few exceptions. The second part of this review highlights these new findings concerning these modifications, and focuses on Ser65-phosphoUb, and its roles in mitophagy and Parkin activation.

## A significantly expanded code

**Figure 2:**
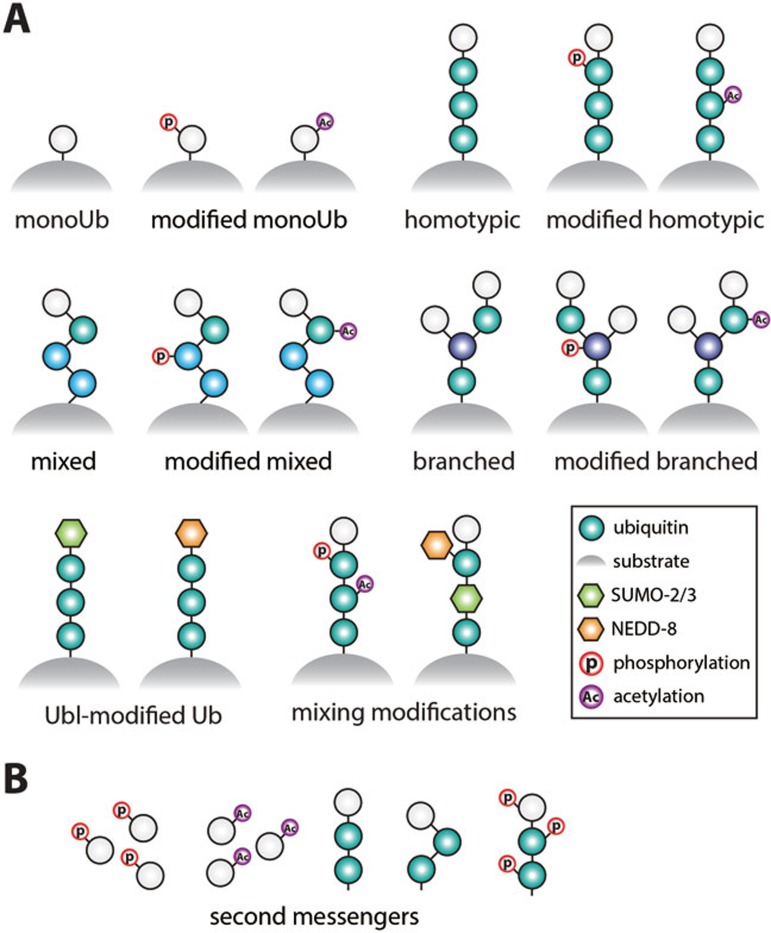
New complexity in the ubiquitin code. **(A)** Conceptual representation of some of the possible ubiquitin-, Ubl (NEDD8, SUMO2/3)- and chemical modifications of ubiquitin. **(B)** Unanchored ubiquitin and ubiquitin chains, with or without modifications, can function as second messengers in cells.

The new layers of ubiquitin modifications significantly alter the features of the ubiquitin code as previously described^19^. Monoubiquitination of proteins can occur with straight or modified ubiquitin, and chains can be homotypic (one linkage type) or heterotypic (Figure 2A). In the latter, chains of one linkage type can be extended by a second type, forming a non-branched structure. Alternatively, a ubiquitin molecule in a chain may be ubiquitinated at multiple Lys residues, forming a 'branched' (also known as 'forked') structure. At this point, we have to assume that all of the ubiquitin moieties, even in complex topologies, could be modified further by acetylation or phosphorylation, or both (Figure 2A).

Considering eight linkage types between ubiquitin molecules, alternative modifications of ubiquitin Lys residues with Ubls or acetyl-groups, and eleven potential phosphorylation sites, this generates an essentially unlimited number of potential combinations.

The different ubiquitin signals can be attached to substrates, and invoke a substrate-specific response. In addition, 'unanchored' ubiquitin or ubiquitin chains exist in cells, and perform second-messenger-like functions^39,40,41^. Monoubiquitin is a prominent component of cell lysates, and once phosphorylated or acetylated, could act as new signaling molecules, in a second-messenger-like fashion (Figure 2B). Indeed, 'free' Ser65-phosphoUb can be detected in cells^42^, and may activate kinase signaling^43^ (see below). Moreover, unanchored polyubiquitin may be DUB-resistant once phosphorylated^44,45^ (see below), and perform functions as a comparably stable independent signaling entity.

## Studying ubiquitination events

**Figure 3:**
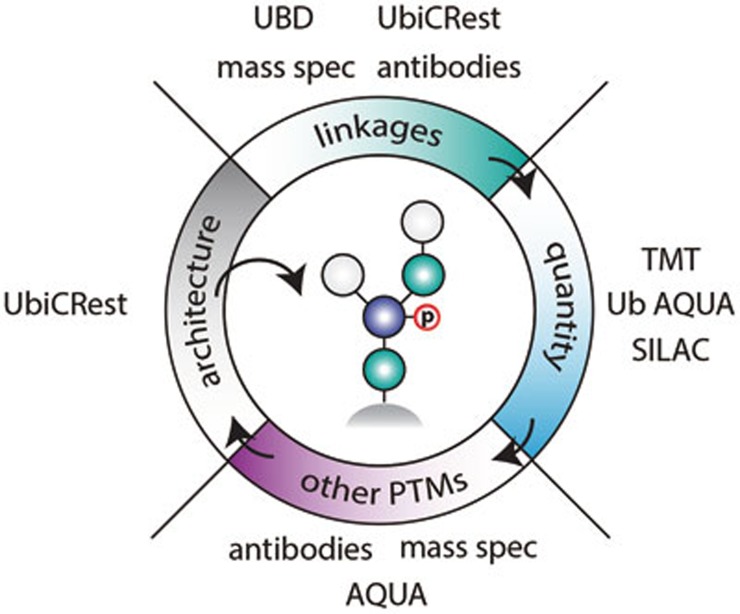
Studying Ub modifications. Linkage-specific UBDs^47,48^, Ubiquitin Chain Restriction (UbiCRest) analysis^46^, linkage-specific antibodies^49,50,51^, a Ser65-phosphoUb antibody^42^ and mass spectrometry^52^ allow for the identification of chain types and ubiquitin modifications. Mass spectrometry enables the quantitation of all ubiquitin linkages in a sample. Relative quantitative techniques include tandem mass tag (TMT) labeling and stable isotope labeling by amino acids in cell culture (SILAC), while absolute quantification (AQUA) strategy determines the exact quantities of each ubiquitin chain type^52^. Similar strategies are available to identify and quantify small molecule and Ubl modifications of ubiquitin.

At this point, it is important to briefly discuss the available tools to understand ubiquitin modifications, which have become quite sophisticated in recent years (Figure 3). New biochemical methods exploit linkage-specific DUBs^46^ and UBDs^47,48^. Linkage-specific antibodies to Met1-, Lys11-, Lys48- and Lys63-linked chains as well as against Ser65-phosphoUb have been developed and provided highly useful reagents^42,49,50,51^. Nevertheless, mass spectrometry has had the most profound impact on studying the ubiquitin code^52^. Quantitative techniques using cell (SILAC) and peptide (TMT) labeling as well as absolute quantification employing labeled ubiquitin peptide standards (AQUA) can be used in conjunction with antibodies enriching ubiquitinated peptides, and enabled many insights into the ubiquitin system. The role of mass spectrometry in studying ubiquitination has been reviewed in-depth recently^52^.

It is important to realize, though, that mass spectrometry-based methods depend on tryptic digestion of ubiquitin and in-depth analysis of the resulting ubiquitin peptides. While this enables annotation and quantification of relative and absolute amounts of certain modifications with the right tools and workflows, it prevents an in-depth understanding of the interplay between modifications. For example, a new area of research dealing with 'branched' ubiquitin chains, in which one ubiquitin molecule is ubiquitinated at multiple sites, is severely compromised by the inability to study all endogenous branched chains. Moreover, it appears quite likely that certain ubiquitin phosphorylation events only exist in the context of particular polyubiquitin chains. It is this information about the hierarchy in modifications that has advanced the histone field, and new methods and tools are essential to achieve a similar degree of insights into the ubiquitin code.

## New insights into ubiquitin chain signaling

Nonetheless, the available methodologies and tools have provided exciting new insights into ubiquitin signaling, and much progress has been made on the enzymatic machinery of the ubiquitin system and functions of differently linked polyubiquitin signals. We previously reviewed the roles of atypical ubiquitin chains^19,25^, however this field has continued to advance rapidly. Below, we provide an update and discuss recent developments for all chain types, and insights into ubiquitin chain architecture. Strikingly, recent data have shaken long-standing dogmas about what constitutes a minimal proteasomal targeting signal, and based on this, we discuss a 'ubiquitin threshold' model for proteasomal degradation.

### Met1-linked 'linear' ubiquitin chains

**Figure 4:**
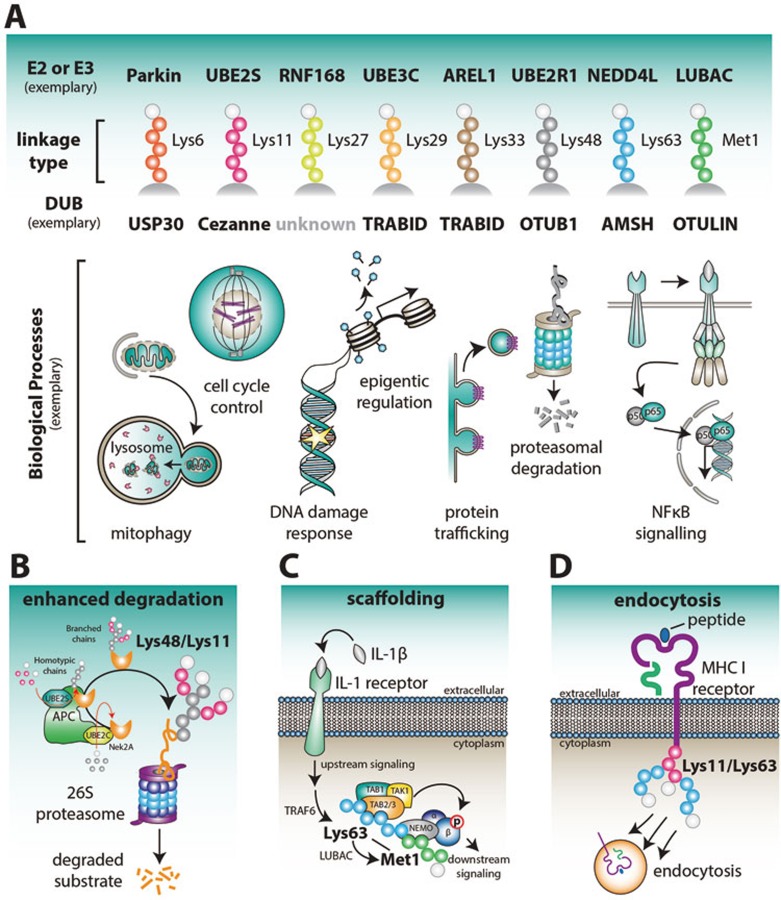
Physiological roles associated with individual chain types. **(A)** A small selection of E2 or E3 enzymes that assemble and DUBs that disassemble ubiquitin chains with linkage preferences is indicated. Below, cartoons illustrate some of the (new) biological processes that particular linkage types have been linked with as discussed in the text. **(B)** APC/C is active during early mitosis and modifies cell cycle regulators such as Nek2A with Lys48/Lys11-linked branched polyubiquitin. In this process, UBE2C first assembles short chains on the substrates, and these are then elongated on each ubiquitin by Lys11-linked polymers. Lys48/Lys11 branched chains enhance proteasomal degradation. **(C)** Mixed or branched Lys63/Met1-linked chains serve as protein scaffolds at immune receptors, such as IL-1 receptors, to promote NF-κB signaling. **(D)** A viral E3 ligase initiates endocytic internalization of the MHC class I receptor through the attachment of mixed or branched Lys11/Lys63-linked ubiquitin chains.

Research on Met1-linked 'linear' ubiquitin chains exemplifies how studies of a particular chain type can advance biology. The field started with the identification of the linear ubiquitin chain assembly complex (LUBAC), which includes an E3 ligase of the RING-between-RING (RBR) family, HOIP, that assembles exclusively Met1-linked polyubiquitin^53^ (Figure 4A). Subsequently, the identification of the Met1-linkage specific UBAN (ubiquitin binding in ABIN and NEMO) domain in NEMO (a component of the Inhibitor-of-κB kinase (IKK) complex), linked Met1-linked chains to NF-κB activation^54,55^. Indeed, LUBAC regulates signaling of tumor necrosis factor (TNF) and related cytokines^56,57^, and Met1-linked chains were found attached to components of the large complexes assembled at their receptors, such as NEMO and RIPK1^56,58^. Indeed, we now understand the molecular basis of LUBAC specificity in exquisite biochemical and structural detail^59,60,61,62^, as well as Met1-linkage specific ubiquitin binding by several small UBDs^55,63,64,65^. In addition, a plethora of genetic models have confirmed important roles of LUBAC in inflammation and immunity^58,66,67,68,69,70,71,72^, and LUBAC components are also mutated or ablated in human inflammatory conditions^73,74^. The physiological roles of Met1-linked ubiquitin chains have been reviewed extensively^75,76^.

Initially, it was unclear which DUB would antagonize Met1-linked polyubiquitin. The ubiquitin-specific protease (USP) enzyme CYLD cleaves Lys63- and Met1-linked polyubiquitin chains with similar activity^54,77^. More strikingly, complete Met1-linkage specificity could be revealed in OTULIN (ovarian tumor (OTU) domain DUB with linear linkage specificity, also known as FAM105B or Gumby)^78,79^ (Figure 4A). OTULIN uses a mechanism of ubiquitin-assisted catalysis, in which Met1-linked polyubiquitin directly regulates the catalytic center of the enzyme^78^. Consistent with the role of Met1-linked chains, OTULIN could regulate NF-κB signaling^78,80,81,82^, but interestingly, OTULIN also affects Wnt signaling^79,83^. A role for Met1-linked chains in this pathway has not been described, and requires further studies. Mechanistically, both CYLD and OTULIN regulate Met1-linked chains by directly interacting with LUBAC, both binding mutually exclusive to the N-terminal PUB domain of the catalytic subunit HOIP^81,82,83,84^. Another OTU DUB, A20, inhibits NF-κB signaling via a distinct route. A20 binds Met1-linked chains via a C-terminal 'A20-like ZnF' domain^64,65^, but its N-terminal OTU domain is unable to hydrolyze this linkage type^85^, and the relevance of A20's DUB function has been questioned^86^. Recent work suggests that upon activation by phosphorylation, A20 is able to hydrolyze Lys63-based scaffolds in cytokine signaling complexes, to which it is recruited via recognition of Met1-linked chains^87^. The interplay between DUBs, and their roles in Met1-linkage ubiquitin and NF-κB signaling are a subject of intense study.

Taken together, Met1-linked ubiquitin chains are now established to be key positive regulators of NF-κB signaling (Figure 4A). Much of the progress made in this area has been facilitated by the identification and characterization of the cellular machineries that assemble, bind and hydrolyze Met1-linked polyubiquitin. This approach serves as a model for research into other, less-well studied, chain types.

### (Re)emerging roles for Lys6-linked polyubiquitin

In contrast to Met1-linked polyubiquitin, physiological roles of Lys6-linked chains are less clear. Lys6-linked chains are not enriched upon proteasome inhibition^16^, potentially indicating non-degradative roles. Global quantitative proteomics studies recently associated this linkage type with two cellular contexts. One study showed that Lys6- as well as Lys33-linkages are upregulated upon UV genotoxic stress^88^. This is interesting since earlier findings concerning this linkage type associated it with the BRCA1/BARD1 E3 ligase complex, an important regulator of the DNA damage response^89,90^. Tools for specific detection of Lys6-linked chains are still missing, and it will be interesting to study the localization and consequences of this modification after DNA damage.

A second set of studies has linked Lys6-linked chains to mitochondrial homeostasis^91,92,93^. Mitophagy, which is described in greater detail in the second part of this review, requires ubiquitination of damaged mitochondria, which is mediated in large part by the E3 ligase Parkin. Like HOIP, Parkin belongs to the RBR family, and assembles predominantly Lys6- and Lys11-, as well as smaller amounts of Lys48- and Lys63-linked chains *in vitro*^91,92^ and on depolarized mitochondria^91,93^ (Figure 4A). Using a ubiquitin replacement strategy or overexpression of ubiquitin mutants, it was shown that mutations of Lys6 and Lys63 (but not Lys48 or Lys11) delayed mitophagy, suggesting that these linkage types invoke specific downstream processes^93,94^.

An intriguing Lys6 preference has also been identified in the DUBs regulating mitophagy and Parkin. USP30, the only DUB constitutively localized to mitochondria, counteracts mitophagy^93,95,96^ and has a preference for Lys6-linked polyubiquitin^44,93^ (Figure 4A). This is noteworthy, USP family DUBs are typically non-specific^97^ (with CYLD being another exception), and the molecular basis for this specificity is unclear. DUBs regulating mitophagy are discussed in more detail in the second part of this review.

Finally, roles of Lys6-linked chains may emerge in other processes such as xenophagy, since Parkin restricts intracellular mycobacteria^98^. Interesting in this context is that a bacterial effector E3 ligase, NleL, from enterohaemorrhagic *Escherichia coli* (EHEC), also assembles Lys6-linkages^99,100^. Its pathophysiological function is unclear, but it is remarkable that bacteria appear to exploit the entirety of the ubiquitin code.

### Lys11-linked chains in the cell cycle and beyond

Together with Met1-linked chains, Lys11-linked chains are the best studied of the atypical chain types, and have been established as an additional proteasomal degradation signal used in particular in cell cycle regulation^101,102^. The Anaphase promoting complex/cyclosome (APC/C) utilizes the only known Lys11-specific E2 enzyme, UBE2S, to assemble this chain type on substrates (Figure 4A)^102^ . Whilst originally regarded as an independent degradation signal, recent data showed that substrates modified with homotypic Lys11-linked chains are poor substrates for the proteasome^103^. Indeed, the predominant form of APC/C-mediated ubiquitination is in the form of branched conjugates, which bind better to the proteasome and are more efficient in promoting protein degradation^103,104,105^ (Figure 4B).

Clear roles for homotypic Lys11-linked polyubiquitin have not been established, however, two DUBs from the OTU family exhibit striking specificity for this linkage type, namely Cezanne/OTUD7B and Cezanne2/OTUD7A (Figure 4A)^85^. Reported physiological roles for Cezanne include regulation of non-canonical NF-κB signaling^106^, T-cell activation^107^, and EGF receptor trafficking^108^, but a clear link to Lys11-linked chains has not been established in these studies.

Cezanne also regulates the adaptation of cells to low oxygen conditions, by regulating hypoxia-inducible factors, HIF-1α and HIF-2α^109,110^. Cezanne knockdown leads to increased Lys11-linked ubiquitination of the transcription factor HIF-1α, and to its subsequent degradation, but this effect could not be rescued by proteasome inhibition. Instead, degradation of HIF-1α in this context likely involves chaperone-mediated autophagy^109^. Cezanne also regulates HIF-2α, in this case through a transcriptional mechanism. HIF-2α is regulated by the transcription factor E2F1, and the loss of HIF-2α caused by Cezanne knockdown can be rescued by overexpression of E2F1^110^. E2F1 degradation may be regulated by a cell cycle and APC/C-dependent mechanism, however, E2F1 loss caused by Cezanne knockdown also could not be rescued by proteasome inhibitors^110^.

Despite these emerging roles, it is striking that receptors for Lys11-linked polyubiquitin have remained obscure. Identification of linkage-specific UBDs will likely fuel our understanding of this chain type, and may reveal new functional context.

### The enigma: Lys27-linkages

Of all the 'atypical' ubiquitin chains, Lys27-linked chains remain the least-well understood and studied. Initial data showing them as a chain type assembled by Parkin^111^ have remained unconfirmed, and a linkage-specific DUB or UBD has proved elusive (Figure 4A).

Two E3 ligases have recently been suggested to assemble Lys27-linked chains in cells. A RING E3 ligase, RNF168, was reported to assemble Lys27-linked ubiquitin chains on histones H2A and H2A.X in cells^112^ (Figure 4A). RNF168 is mechanistically interesting as it is one of the few RING E3 ligases with additional UBDs that may bind and orient an 'acceptor' ubiquitin and potentially furnish the ligase with linkage selectivity. Consistent with known roles for RNF168, Lys27-linked chains were upregulated by a DNA damaging reagent that induce DNA double-strand breaks, and shown to recruit DNA damage repair factors, such as p53 binding protein 1 (53BP1), to DNA damage foci. Together, these data imply that Lys27-linked chains may serve as scaffolds for protein recruitment in the DNA damage response (DDR) (Figure 4A).

A second E3 ligase suggested to assemble Lys27-linked chains is HECT domain and ankryin repeat containing E3 ubiquitin ligase 1 (HACE1). Using ubiquitin mutants, two papers independently reported that HACE1 modifies its substrates Optineurin (OPTN, see below) and Y-box binding protein 1 (YB-1), with Lys27-linked polyubiquitin^113,114^. The reported roles for Lys27-linked modification in these instances are regulation of protein secretion (YB-1) and increase of autophagic flux (OPTN).

All three reports suggest that Lys27-linked chains act to recruit proteins, suggesting the presence of specific UBDs, and indeed, UBDs from DDR components interact well with Lys27-linked diubiquitin^112^. With these interesting leads, Lys27-linked chains are likely to become less enigmatic in the near future.

### New roles for Lys29-linked chains in proteasome regulation and epigenetics

For Lys29- and Lys33-linked chains the first set of linkage-specific proteins for assembly, recognition and hydrolysis is now known, enabling genetic approaches and detailed studies into chain function. Lys29-linked chains are assembled by the HECT E3 ligase KIAA10/UBE3C that assembles K29- and K48-linked polyubiquitin chains^115,116,117^ (Figure 4A), and associates with the 26S proteasome^115,118^. Interestingly, while the yeast orthologue Hul5 also resides on proteasomes, it assembles Lys63- rather than Lys29-linked chains^119^. In human cell lines, proteasome inhibition results in enrichment of Lys29-linked chains^16^, suggesting that Lys29-linked chains can be a proteasomal degradation signal. However, UBE3C modifies the proteasome ubiquitin receptor Rpn13 with Lys29-linked chains in response to proteasomal stress, to prevent further substrate engagement^118^. This could be an alternative reason why this chain type is enriched in proteomics studies with inhibited proteasomes.

Beyond proteasomal degradation, new insights into Lys29-linkages emerged from the identification of Lys29/Lys33-specificity in the deubiquitinase TRABID (Figure 4A). TRABID contains an extended linkage-specific OTU domain^120,121^, and in addition, an N-terminal UBD module with three Npl4-like zinc finger (NZF) domains that recruit TRABID to Lys29/Lys33-linked polyubiquitin chains in cells^116,117,121^. Initial data on TRABID linked it to Wnt-mediated transcription^122^, and a recently generated mouse knockout model identified its roles in epigenetic regulation^123^ (Figure 4A). In this model, Trabid regulates transcription of a set of interleukins (IL12 and IL23) downstream of Toll-like receptor (TLR) signaling, yet in an indirect way. It was reported that a target of Trabid in this pathway is the histone demethylase Jmjd2d, which it appears to deubiquitinate and stabilize, so that Jmjd2d can act on the interleukin gene promoters to release repression^123^. Indeed, loss of Trabid leads to modification of Jmjd2d with Lys29- and Lys11-linked chains, linking these chain types to functional outcome.

### Lys33-linked polyubiquitin in intracellular trafficking

The HECT E3 ligase AREL1 was shown to make Lys33- as well as Lys11-linked polyubiquitin chains, and was used to generate Lys33-linked chains in large quantities for the first time^116,124^ (Figure 4A). AREL1 has been scarcely studied. In a single report, a role as a negative regulator of apoptosis was suggested, whereby AREL1 may ubiquitinate antagonists of inhibitor of apoptosis (IAP) proteins, including SMAC, HtrA2 and ARTS^125^. The modification is suggested to lead to protein degradation. However, chain type(s) involved in cells were not studied; *in vitro* reconstitution assays showed predominantly Lys33-linkages on the three substrates as a result of AREL1-catalyzed ubiquitination^116^.

Lys33-linked chains have been implicated in various biological processes^25^, and more recently, in post-Golgi membrane protein trafficking^126^ (Figure 4A). The BTB domain-containing adapter protein, KLHL20, works with a Cullin-3 E3 ligase complex to catalyze Lys33-linked polyubiquitination of Coronin 7(Crn7), which promotes its recruitment to the *trans*-Golgi network (TGN), indicating roles in anterograde trafficking^126^. These roles for Lys33-linked chains were revealed using an elegant ubiquitin replacement strategy, in which cellular ubiquitin is replaced with a K33R ubiquitin mutant. However, similar to much of the other research on atypical chains, most insights into Lys33-linkages are based on isolated studies and require follow-up research to solidify specific roles.

### Lys48-linked chains ― re-defining a proteasomal degradation signal

As mentioned above, Lys48-linked chains are the most common chain type and target proteins for proteasomal degradation (Figure 4A). Elegant biochemical studies by the Pickart lab showed that proteins require at least a tetraubiquitin chain to be efficiently targeted to the proteasome^127^. This tetraubiquitin dependence was the dogma in the field for the last decade, despite subsequent structures of the proteasome lid that illuminated its ubiquitin chain receptors and DUBs, and did not quite explain the tetraubiquitin requirement^128,129,130^. Moreover, the ubiquitination pattern of efficiently degraded proteins such as Cyclin B1 revealed multiple short chains of various linkages, rather than singular long chain^131^. Also, the models did not explain how some proteins can use single Lys48-linked chains for non-degradative means^132^, although protection by UBDs likely plays a role^133^.

Interesting biochemical and biophysical studies have started to re-define the minimal requirements for proteasomal degradation^134^. In this system, a substrate modified with four monoubiquitin modifications was not degraded. Interestingly, two diubiquitin modifications were a better degradation signal as compared to a single tetraubiquitin chain^134^. More detailed studies along these lines, controlling for chain type, chain length and chain position on a substrate, appear essential to finally understand what constitutes the perfect proteasomal degradation signal. Differences in these three parameters could easily lead to distinct degradation kinetics, and a new layer of regulation, as suggested in the initial study^134^.

### Lys63-linked chains and the fight for the crown in inflammatory signaling

The second 'canonical' Lys63-linked chain type has many well-studied (and extensively reviewed) non-degradative roles^19,24,25^. Most notoriously, this linkage type was at the heart of a debate in inflammatory signaling and NF-κB activation, that started when Met1-linked chains were found to be important in the underlying signaling cascades (Figure 4A). Specific UBDs for each chain type are present in the cascading kinase complexes ― the 'upstream' TAK1 kinase complex contains Lys63-linkage specific UBDs in TAB2 and TAB3^135,136,137^, while the 'downstream' IKK complex encodes Met1-linkage specificity in the NEMO UBAN domain^54,55,138^ (see above).

The debate has been elegantly resolved by biochemical findings, which clearly show that Lys63-linked chains are modified with Met1-linked chains in mixed or branched architectures^139^ (Figure 4C). LUBAC binds to polyubiquitin of various compositions^57^, and the mechanism of HOIP requires binding of an acceptor ubiquitin that could be part of an existing chain^61^. Heterotypic or 'hybrid' chains were revealed by using linkage-specific DUBs in a method called 'ubiquitin chain restriction analysis'^46^, where a Lys63-linkage specific DUB released blocks of Met1-linked polyubiquitin, and a Met1-linkage specific DUB removed predominantly the high-molecular weight portion of the polyubiquitin smear^139^, showing that the chains started with Lys63-linkages on the substrate.

Mixed-linkage Lys63/Met1-linked chains seem like the solution to recruit distinct kinase complexes, and may resolve a debate about the specificity of NEMO (Figure 4C). In addition to its UBAN domain, NEMO contains a zinc-finger UBD module that was reported to prefer Lys63-linkages^140,141^. Two UBDs with distinct specificities may indeed target the IKK complex to Lys63/Met1 linkage junctions within the polymer.

### The rise of 'invisible' branched and mixed chains

As exemplified by Lys63/Met1 hybrid chains, mixed and branched chains could be charged with new signaling information. Indeed, all the above-mentioned new roles for atypical chains may rely on interplay with other linkage types. Jmjd2d ubiquitination involves Lys11- and Lys29-linked chains^123^, and HECT and RBR E3 ligases often assemble a defined subset of linkages (NleL, Lys6/Lys48^99^; HACE1, Lys27/Lys48^113^; UBE3C, Lys29/Lys48^115^; AREL1, Lys33/Lys11^116^). Consistently, when enriched from cells, Lys29-linked chains are predominantly part of heterotypic polyubiquitin^117^.

The power of branched chains to provide new complexity to the system is highlighted by research on the APC/C, which assembles Lys48/Lys11-branches that are efficient proteasomal degradation signals^103,104,105^ (Figure 4B), or viral E3 ligases that assemble branched chains to initiate endocytosis of host proteins^142,143^ (Figure 4D). In contrast, earlier reports indicated that some types of branched chains lead to non-degradable ubiquitin structures and proteasome stalling^144^, and are actively prevented in cells^145^. We think that both possibilities are likely, and much of the outcome will depend on how the (proteasomal) DUBs can handle complex chain architectures.

Additional roles for chain branching are likely to be identified in the coming years. However, as also described above, we are essentially blind to these modifications, as the methods to distinguish chains generally do not report on chain architecture. Mass spectrometry can only cleanly identify those branches in which two neighboring Lys residues are modified, such as Lys29/Lys33^15^ or Lys6/Lys11^144^. The majority of branched conjugates ― there are 28 different combinations in which one ubiquitin can be modified on two Lys or Met1 residues ― cannot be identified or quantified easily. Current methods rely on limited proteolysis by trypsin and hence suffer from systematic errors^146,147^. It is also not clear, whether higher-order branched ubiquitin (ubiquitin ubiquitinated on more than two Lys residues) exists in cells. To study this complexity in chain architecture, new methods need to be developed that enable analysis of the branched ubiquitin pool of a cell.

## A 'ubiquitination threshold' model for proteasomal degradation

**Figure 5:**
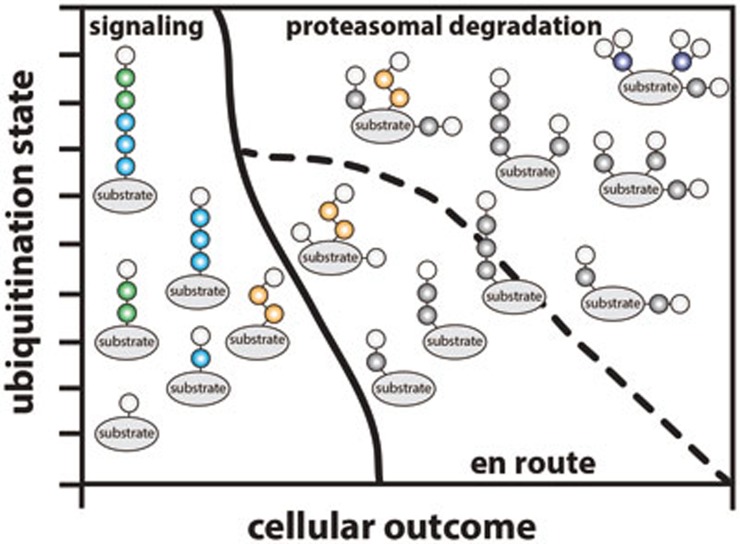
A 'ubiquitin threshold' model for proteasomal degradation. Substrate ubiquitination can result in two general outcomes, cellular signaling or proteasomal degradation. Recent evidence supports a model in which multiple short chains (e.g., diubiquitins) or branched ubiquitin are better degradation signals as compared to a single Lys48-linked tetraubiquitin. These findings also suggest that non-degradative ubiquitin signals could be modified into degradative signals through addition of short and/or branched ubiquitin chains to substrates.

The new insights into individual chain types, new rules for proteasomal degradation, and rise of branched and mixed linkage chains, support a 'ubiquitination threshold' model for proteasomal degradation (Figure 5). Clearly, the main task of the ubiquitin system is proteasomal degradation, and the proteasome can degrade a range of substrates in various contexts, not all of which depend on (poly)ubiquitination^148^. With the new data on degradation signals (see section on Lys48-linkages above) the number of distinct signals to initiate degradation increases further; multiple modifications with short Lys48-linked chains, or branched structures with Lys11- or Lys48-linkages are clearly efficient signals. In other words, a protein performing a ubiquitin-mediated, non-degradative task (e.g., modified with a Lys33-linked chain), once modified on another nearby substrate Lys, or modified on the ubiquitin chain itself to create a branched structure, could with little effort be destined for proteasomal degradation. Indeed, the possibility of branching would enable any non-degradative chain type to become a degradation signal. The transition from defined degradation signals (Lys48-linked tetraubiquitin) to a 'ubiquitination threshold' model, where the amount of polyubiquitin rather than the type is important, may explain a lot of existing biochemical and proteomic data. For example, the most abundant E2 enzymes such as those of the UBE2D family, appear to modify proteins on random sites with short chains of many types^144^, and Cyclin B degradation is facilitated by multiple short chains of various types^131^.

In contrast, specialized degradative E3 ligase systems such as SCF E3 ligases, utilize the Lys48-specific E2 enzyme UBE2R1, which assembles chains of medium-lengths (on average 3-6 ubiquitin molecules) on model substrates *in vitro*^149^ to serve as efficient 'canonical' degradation signals (Figure 4A).

Another important component in this context is the fact that ubiquitination is highly dynamic. DUBs regulate which chain types are assembled, and how the ubiquitination status of a protein will be shaped. Many E3 ligases exist in conjunction with DUBs, and could perform essential chain editing functions^13^. Interesting in this context is also the idea that the length and dynamics of ubiquitin chains could determine their susceptibility to DUBs and determine the relative stability of a ubiquitin signal^150,151^. To conclusively discuss what defines the 'perfect' proteasomal degradation signal, we need to be able to study chain length and chain branching in more detail.

Conceptually, the idea that 'signaling' pools of ubiquitinated proteins could be very similar to 'proteasome-targeted' pools, provides challenges to studying the roles of the signaling chain types. It is the former, likely very small pool of proteins, that will reveal the signaling roles of atypical chains. Especially for the studies of lowly abundant atypical chains, the search for these 'needles-in-the-haystack' will continue to be challenging, but as discussed above, the 'magnets' to extract them and uncover their context are becoming stronger.

## Alternative ubiquitin modifications: a second layer in ubiquitin signaling

So far, we have discussed ubiquitin chains, and the many forms and functions that this first layer of the ubiquitin code entails. A second layer is provided when ubiquitin is modified with other kinds of modifications, which could include conjugation by other Ubl modifiers, or small chemical modifications such as phosphorylation or acetylation. The remainder of the review will discuss these modifications, which present new complexity, yet also new functionality and ways to regulate the ubiquitin system.

## SUMOylated and NEDDylated (poly)ubiquitin

SUMOs constitute the best studied Ubl modification system, which competes with ubiquitination for modifications of Lys residues. As compared to the ubiquitin system, the relative simplicity of the SUMO system with a single E1, a single E2 and a small number of E3 enzymes has enabled a comprehensive set of mechanistic studies^152^. Physiological roles of SUMOylation have emerged in many biological processes, in particular in transcription, DNA repair, and various stress responses^153^. Interestingly, SUMO also forms chains, and SUMO-targeted ubiquitin ligases ('StUbLs') target SUMO chains for ubiquitination^154^.

In addition to ubiquitinated SUMO chains, and more relevant to this review, is the recent findings by proteomics studies that ubiquitin can be SUMOylated. Multiple ubiquitin Lys residues (Lys6, Lys11, Lys27, Lys48, and Lys63) can be targeted for SUMOylation, implying a potentially complex web of intertwining modifications^26,27,28^. While the functional roles of these SUMO-ubiquitin chain types are still ambiguous, SUMOylation of ubiquitin Lys6 and Lys27 is upregulated in response to heat shock and proteasome inhibition^28^, providing a starting point for further investigation. Mechanistically, ubiquitin SUMOylation is also interesting. Typical SUMOylation motifs, such as a SUMO E2 binding site or a SUMO-interacting motif^153^, are missing in the well-folded ubiquitin molecule, and hence, a ubiquitin-targeted SUMO ligase ('UbtSL') should be responsible, but remains to be identified.

It is possible that other Ubl modifiers, such as NEDD8 or ISG15, also modify ubiquitin and ubiquitin chains. With overexpression of NEDD8, NEDDylated ubiquitin can be readily observed^155^, however, overexpression leads to the non-physiological use of NEDD8 by the ubiquitination machinery^156^. It is unclear to what extent NEDD8 modifies ubiquitin under physiological conditions.

The idea that ubiquitin and polyubiquitin could be modified by Ubl modifications adds significant complexity to the system, and highlights the crosstalk between modifications (Figure 2).

## Ubiquitin phosphorylation and acetylation

The modifications of amino acids by small chemical groups such as phosphate, methyl and acetyl groups, sugars or lipids are highly abundant and regulate many fundamental processes in biology. Most comprehensively studied are protein phosphorylation and acetylation, and their characterization was fueled by peptide-level affinity enrichment strategies that led to discoveries of tens-of-thousands of modified sites in proteomes^157^. Due to the wealth of proteomics datasets, it is not uncommon to find your protein of interest to be phosphorylated or acetylated or both. The common challenge, however, is to understand whether a modification is a genuine way to alter a protein's function, or whether it is a side reaction without relevance. Most available proteomics datasets provide steady-state snapshots without information on relative abundance (which is commonly < 1% of a protein pool), and often without dynamic information (i.e., changes of a modification upon a stimulus or over time etc.), making assessment of the 'importance' of particular modifications difficult.

It should not be a surprise that phosphorylation and acetylation extend to ubiquitin and Ubl modifiers, and while this information has been available for some time, the first studies into their physiological relevance have only surfaced in 2014. The identification of functionally important phosphorylation of ubiquitin at Ser65, performed by PTEN-induced protein kinase 1 (PINK1) during mitophagy (discussed below), has triggered enormous excitement and research into this and other chemical modifications of ubiquitin. Importantly, both types of modifications, Lys acetylation and Ser/Thr/Tyr phosphorylation, affect charge and surface properties of the ubiquitin molecule ― particularly important since most surfaces of ubiquitin are functional in some contexts of the ubiquitination machinery or during ubiquitin binding. Below, we review what is known about ubiquitin acetylation and phosphorylation, as well as future directions and open questions.

## Codes collide with acetylated ubiquitin

Lys acetylation competes with ubiquitin chain formation and hence impacts chain architecture. AcUb with modifications on Lys6 or Lys48 is readily detected in cells^29^, but acetylation also occurs on other Lys residues (Figure 1B). Acetylated ubiquitin is readily available via non-natural amino acid incorporation, and this was used to assess the impact of AcUb on the ubiquitination machinery^31,158^. Lys6-AcUb and Lys48-AcUb did not interfere with E1-mediated E2 charging, however, the discharge of ubiquitin onto substrates was inhibited in a variety of E2/E3 assembly reactions^31^.

Importantly, the impact of Lys acetylation may not only extend to the modified Lys, but may also affect chain assembly or disassembly at nearby Lys residues. An intriguing example is the E2 enzyme UBE2S, which utilizes Lys6 in assembly of Lys11-linked polyubiquitin chains^159,160^ or the ubiquitination of MHC class I with Lys11/Lys63 branched chains, which also requires a functional Lys6 residue^142^. Hence, the effects of ubiquitin Lys acetylation may be manifold.

As discussed above, histones in nucleosomes are amongst the most modified proteins in eukaryotes in terms of density and variety of post-translational modifications^20^. Importantly, Lys6- and Lys48-AcUb has been identified attached to histones in cells^31^. An 'acetyl-mimetic' ubiquitin mutation (K6Q) was found to stabilize monoubiquitinated histone H2B^31^. Further research needs to address whether and how AcUb extends the histone code. It is tempting to speculate that the histone acetylation and deacetylation machineries also serve to modify the nearby attached ubiquitin molecules ― after all, histones are the most abundantly ubiquitinated proteins in our cells.

Despite this emerging context, the enzymes responsible for ubiquitin acetylation and deacetylation, as well as functional role(s) of acetylated ubiquitin, are unknown. Recombinantly produced site-specific AcUb^31,158^ should enable identification of deacetylases, and may help to reveal AcUb-binding domains. Yet, as for other ubiquitin modifications, it is paramount to identify the acetyl-transferases that mediate ubiquitin acetylation to place AcUb into its cellular context.

## Increased complexity with phosphoUb, and links to Parkinson's disease

As with acetylation, evidence of ubiquitin phosphorylation on eleven sites was available in proteomics databases (Figure 1). Amongst these, Ser65-phosphorylation of ubiquitin is lowly abundant under steady state conditions, but dramatically increased when mitochondria are depolarized, providing cellular context of its regulation. These findings have initiated the emerging research field of ubiquitin phosphorylation. Whether and which kinases phosphorylate ubiquitin site-specifically is still unclear, however, the work on the PINK1/Parkin system exemplifies how such discoveries can come at a rapid pace.

In a nutshell, ubiquitin was identified as the main substrate for the protein kinase PINK1, which has been intensely studied due to its genetic links to autosomal recessive juvenile Parkinsonism (AR-JP), an early onset form of Parkinson's disease (PD). The discovery of ubiquitin phosphorylation originated from (a) the genetic link of PINK1 and Parkin, an E3 ligase also mutated in AR-JP, (b) cell biological studies that placed PINK1 upstream of Parkin in mitophagy, (c) the search for the PINK1 substrate that acts as Parkin receptor on mitochondria and (d) the search for a mechanism of Parkin activation. This and the subsequent discoveries surrounding Ser65 phosphorylation are described below.

## Mitophagy ― a new playground in ubiquitin research

**Figure 6:**
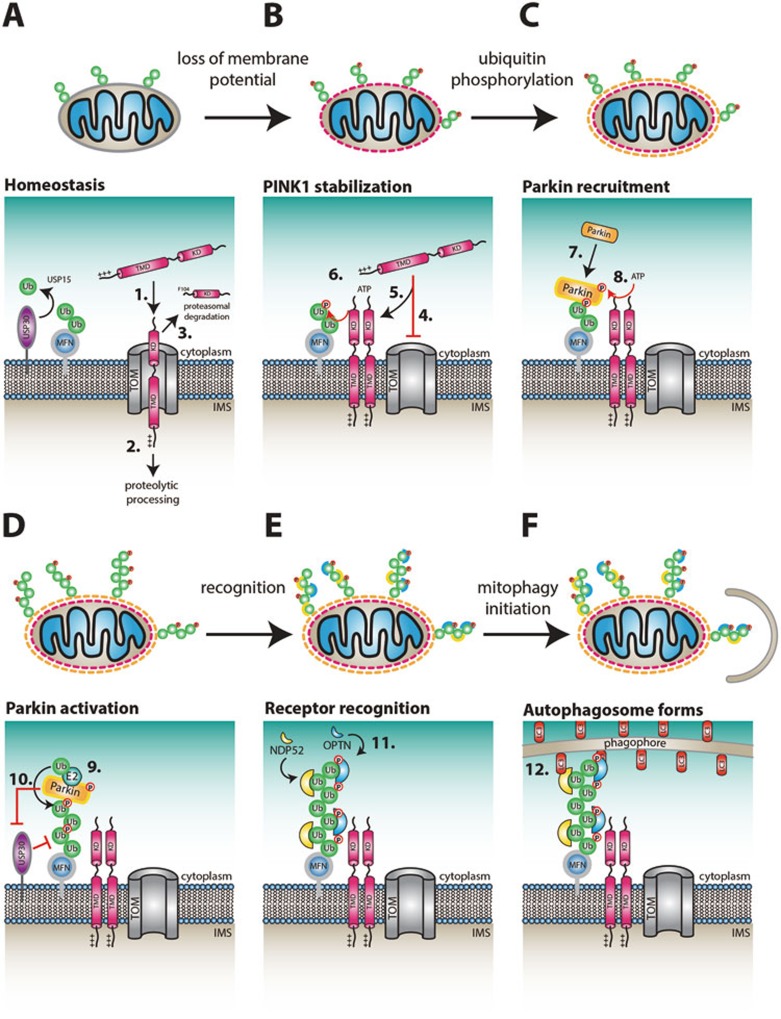
Ser65-phosphorylation of ubiquitin in mitophagy signaling. **(A)** Under normal growth conditions, the TIM/TOM complex continually imports PINK1 into mitochondria (step 1). Upon entry, PINK1 undergoes proteolytic processing by the protease PARL (step 2) and is exported and degraded by the N-end rule pathway (step 3). USP30 controls the basal levels of mitochondrial ubiquitination. **(B)** Loss of mitochondrial membrane potential inhibits PINK1 import and proteolytic cleavage (step 4), leading to insertion of its transmembrane domain into the outer mitochondrial membrane (OMM) (step 5). PINK1 phosphorylates ubiquitin on mitochondrial proteins such as mitofusins (step 6). **(C)** Ser65-phosphoUb recruits Parkin to damaged mitochondria (step 7), and Ser65-phosphoUb binding releases the Parkin Ubl domain and enables its phosphorylation by PINK1 (step 8). **(D)** Ser65-phosphoUb binding and phosphorylation activate Parkin which subsequently ubiquitinates OMM proteins, and the newly incorporated ubiquitins are further phosphorylated by PINK1 (step 9). Parkin-mediated ubiquitination of USP30 facilitates its proteasomal degradation (step 10). **(E)** Mitophagy receptors NDP52 and OPTN bind to ubiquitinated mitochondrial proteins via their UBDs (step 11). **(F)** NDP52 and OPTN recruit the autophagy machinery to mitochondria (step 12). The phagophore engulfs mitochondria and fuses with the lysosome to degrade and recycle its contents.

Mitophagy is the process by which cells selectively destroy damaged mitochondria in an autophagy-like fashion^161^ (Figure 6). Mitochondria constitute a dynamic organelle network within cells, and are in constant flux with ongoing fusion and fission events. When parts of mitochondria are damaged, e.g., through accumulation of misfolded proteins or loss of membrane potential, the damaged parts of the mitochondria are tagged, isolated and subsequently destroyed^161^. A variety of mechanisms for destruction are in place, including autophagosomal degradation (mitophagy), but also proteasomal degradation and smaller-scale lysosomal degradation via vesicular routes^162^. Mitophagy has predominantly been studied by global depolarization of all cellular mitochondria, using uncoupling agents such as CCCP. Based on cell biological work, the mitochondrial protein kinase PINK1 was shown to be a key player in the process. PINK1 is constitutively degraded through proteolytic cleavage of its transmembrane domain by the transmembrane protease PARL^163^, and subsequent ubiquitination and proteasomal degradation through N-end-rule E3 ligases^164^ (Figure 6A). Upon mitochondrial damage, such as loss of membrane potential, PARL-dependent cleavage of PINK1 is inhibited and PINK1 accumulates on damaged mitochondria^165^ (Figure 6B). PINK1 stabilization and catalytic activity lead to rapid accumulation of the E3 ligase Parkin on the outer mitochondrial surface^166^ (Figure 6C). Many PINK1 substrates and Parkin receptors have been proposed^167,168^, but an important breakthrough came with the realization that PINK1 targets Parkin itself, phosphorylating it on Ser65 in the N-terminal Ubl domain^169,170^ (Figure 6C).

Seminal studies then showed that PINK1 also phosphorylates ubiquitin at Ser65^44,91,171,172,173^ (Figure 6C). Ubiquitin and the Parkin Ubl domain are highly similar in structure and both contain Ser65 in a structurally identical position^172,173^. In addition, mass spectrometry experiments comparing wild-type and PINK1- knockout cells^91,171^ or analyzing reconstituted Parkin reactions^44^ revealed a substantial increase for the Ser65-ubiquitin phosphorylation site only. Ser65-phosphoUb is barely detectable in cells lacking PINK1, and < 0.1% of total ubiquitin in wild-type cells without mitochondrial depolarization, but rises to more than 2% of the total ubiquitin pool in cells after CCCP-induced depolarization^91,172^. On mitochondria, ∼20% of all ubiquitins are phosphorylated at Ser65 after CCCP treatment^91,94^. The phosphorylated ubiquitin acts as a recruitment platform both for Parkin^91,94,171,172,173,174,175^ (Figure 6D), and for autophagy adaptors that initiate mitophagy^176,177^ (Figure 6E and 6F). Discovery of PINK1 as the first ubiquitin kinase enabled not only wide-ranging studies into cell biology, but also into ubiquitin biochemistry and structure.

## Impact of ubiquitin phosphorylation on ubiquitin structure and enzymology

**Figure 7:**
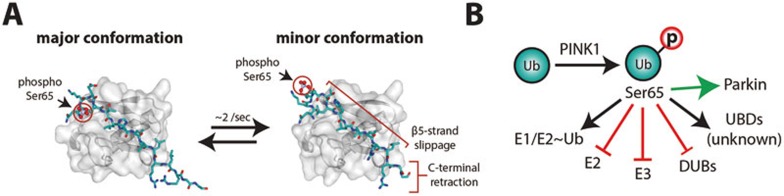
Structural and functional consequences of Ser65-phosphorylation of ubiquitin. **(A)** Ser65 phosphorylation generates a dynamic equilibrium between two ubiquitin conformations. A major conformation structurally resembles unmodified ubiquitin, but has altered electrostatic potential. The minor conformation has a retracted C-terminus induced by slippage of the β5-strand. The images show the differing region in stick-representation embedded in the remaining ubiquitin core under a surface. The phosphorylated Ser65 is indicated. **(B)** Ser65-phosphorylation of ubiquitin has neutral, loss-of-function and gain-of-function effects on components of the ubiquitin system. E1 and E2 charging is largely unaffected; however, E2 discharging and chain elongation mediated by a subset of E2 and E2/E3 chain assembly systems are inhibited. A substantial numbers of DUBs have reduced activity against Ser65-phosphoUb chains. Receptors recognizing Ser65-phosphoUb are unknown. Ser65-phosphoUb allosterically activates the E3 ligase Parkin and may activate kinase signaling towards Rab GTPases.

Recombinant PINK1 enables access to Ser65-phosphoUb for biochemical and structural studies^178^. Surprisingly, Ser65 phosphorylation changes ubiquitin structure in novel ways, generating two ubiquitin conformations that are in dynamic equilibrium^44^ (Figure 7A). The predominant 'major' conformation resembles the common ubiquitin structure that has been observed more than 300 times in the Protein Data Bank (PDB)^179,180^, but has important altered surface potential due to the phosphorylation on Ser65. A second, 'minor' conformation of ubiquitin had not been observed previously. In this conformation, the last β-strand and C-terminal tail of ubiquitin are withdrawn into the molecule, altering many surface properties including the Ile44 patch and restricting the C-terminus for chain assembly^44^ (Figure 7A). The relevance of the new conformation is not clear yet, but it seems likely that unknown binding partners could stabilize and recognize it specifically.

Moreover, striking effects of Ser65-phosphoUb on the ubiquitin system have been revealed (Figure 7B). Ser65-phosphoUb is charged normally onto E2s by the E1 enzyme, but discharge into polyubiquitin chains is inhibited in several systems, most notably the UBE2N/UBE2V1-mediated generation of Lys63-linked chains^44,45^. Moreover, some RING E3 ligases, such as TRAF6, no longer assemble polyubiquitin chains with the UBE2D-family enzyme if only Ser65-phosphoUb is provided in the reaction^45^. Free Ser65-phosphoUb only accounts for a small percentage of the total ubiquitin pool though, and an impact on E3 ligase function seems unlikely.

However, interesting data suggest the existence of Ser65-phosphoUb-dependent protein kinase(s)^43^. In a search for new PINK1-dependent phosphorylation events, small GTPases of the Rab family were discovered as targets for phosphorylation, however, the protein kinase for Rab proteins was not PINK1^43^. It is a likely scenario that in this instance, Ser65-phosphoUb, either on mitochondria or perhaps in its free form (second messenger role, Figure 2B), acts to activate a protein kinase for Rab proteins. The Parkinson's disease kinase LRRK2 was reported to target a subset of Rab proteins^181^, yet an influence of Ser65-phosphoUb on LRRK2 was not tested.

More prevalent are the roles of Ser65-phosphoUb on mitochondria, where ubiquitin chain modifications are recognized by (phospho)Ub-binding proteins to initiate mitophagy, or targeted by DUBs or ubiquitin phosphatases to restrict mitophagy (see below). The most prominent and best-understood role of Ser65-phosphoUb on mitochondria is to recruit and activate Parkin.

## Regulation of Parkin activity

**Figure 8:**
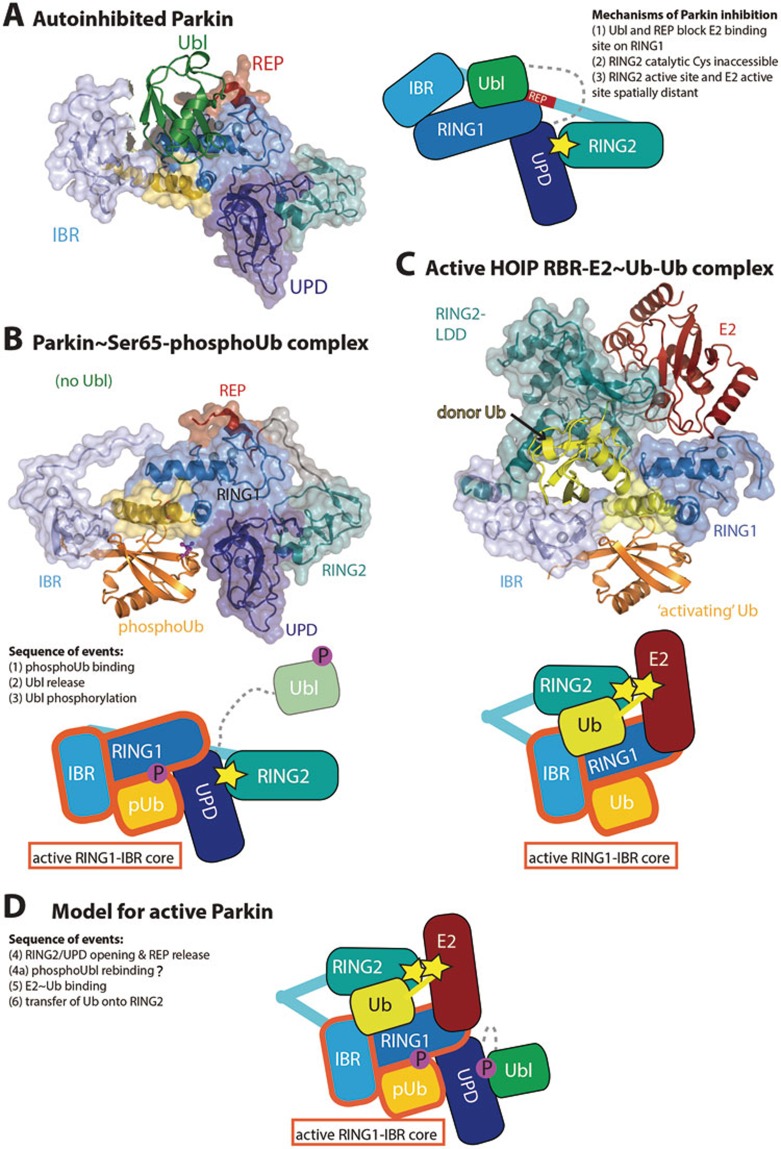
Insights into Parkin activation. **(A)** Structure of autoinhibited full-length Parkin (PDB ID: 4K95,^185^). Domains are colored in green (Ubl), dark-blue (UPD, also known as RING0), blue (RING1), light-blue (IBR), cyan (RING2) and red (REP). The mechanisms of Parkin inhibition are listed in the schematic figure. **(B)** Complex structure of Parkin bound to Ser65-phosphoUb. Coloring as in **A** with Ser65-phosphoUb in orange. Conformational changes in RING1 and IBR domain form the Ser65-phosphoUb-binding site. The Ubl domain was not included in the crystallized construct. **(C)** Structure of a HOIP RBR module bound to ubiquitin-charged E2 (yellow/red) and an extra, 'activator' ubiquitin. The structures in **B** and **C** are shown side-by-side to point out the identical active RING1-IBR module, indicated in the cartoon. Importantly, RING2 is juxtaposed to the E2 active site to receive ubiquitin. **(D)** The model of HOIP in **C** may indicate what active Parkin could look like. In this model, the RING2-UPD interface has been opened, and RING2 now sits atop RING1/IBR to receive ubiquitin. The hydrophobic surface on the UPD that was occupied by RING2, could be covered by rebinding of the phosphorylated Ubl domain^188^.

Parkin recruitment to mitochondria leads to a rapid and striking increase in mitochondrial ubiquitination^91^, however, Parkin also has roles outside of mitochondrial maintenance, e.g., in the restriction of *Mycobacteria*^98^ and in cell signaling processes^182^. Parkin is primarily cytosolic, and its role in AR-JP has resulted in many studies of its activity and of its substrates. Like HOIP, Parkin belongs to the RBR family of E3 ligases that act via a covalent ubiquitin-bound intermediate^183^. Ubiquitin-charged E2 enzymes such as UBE2D or UBE2L3 are bound by the RING1 domain in Parkin, and transfer their ubiquitin not directly to a substrate Lys, but first onto a Cys residue in the RING2 domain (Figure 8). From there, substrates are modified; as mentioned above, Parkin predominantly generates Lys6-, Lys11- and to a lesser extent, Lys48- and Lys63-linked polyubiquitin, in chains of unstudied architecture^91,92^.

Importantly, structural and biochemical work has revealed that Parkin is autoinhibited in several ways (Figure 8A). An N-terminal Ubl domain in Parkin is autoinhibitory^184^, but subsequent crystal structures of Parkin with and without the Ubl domain revealed a greater extent of autoinhibition^185,186,187^. Indeed, both the Ubl domain, and the repressor element of Parkin (REP) situated in the In-Between-RING (IBR) and RING2 domain linker, block the E2 binding site of the RING1 domain. Moreover, the catalytic Cys residue in the Parkin RING2 domain is inaccessible, as it is located at a tight, hydrophobic interface with the Unique Parkin Domain (UPD also referred to as RING0). Even if the E2 could bind to the RING1 domain, modeling suggests that the RING2 and E2 catalytic centers would be on opposite faces of the protein. These findings illustrated the need for significant conformational changes to activate Parkin^185,186,187^.

Strikingly, the required conformational changes can be invoked by Ser65-phosphoUb. Robust ubiquitination by Parkin is observed in presence of PINK1, but purified Ser65-phosphoUb can bypass the requirement for PINK1. Curiously, Parkin cannot utilize Ser65-phosphoUb in chain assembly reactions^44,91^, but instead uses it as an allosteric regulator^172^. The molecular detail of this became clear from a crystal structure of Parkin in complex with Ser65-phosphoUb^188^ (Figure 8B), which showed how a cryptic Ser65-phosphoUb-binding site not present in autoinhibited Parkin, is formed through straightening of a RING1 helix, inducing movement of the IBR domain. In the complex structure, Ser65-phosphoUb is cradled by the UPD, RING1 and IBR domains, explaining the nanomolar affinity^188^ (Figure 8B). Conformational changes of the IBR domain has multiple effects, and destabilizes the REP-mediated RING1 repression, but more importantly, also leads to a release of the Ubl domain from the Parkin core^188,189,190,191^. Together, this enables binding of the charged E2^172,189^. The complex structure has not lost all of its autoinhibited character as the RING2:UPD interface continues to block access to the RING2 active site^91,188^. Further conformational changes are hence required to fully unblock Parkin.

What such conformational changes might look like was revealed recently in a structure of the HOIP RBR domain module trapped in a catalytically active conformation bound to a ubiquitin-charged E2 enzyme^62^ (Figure 8C). In this structure, the RING2 domain is juxtaposed to the IBR domain to receive ubiquitin from the E2 active site. Excitingly, the IBR domain adopts an identical position with respect to the RING1 domain as observed in the Ser65-phosphoUb-bound Parkin structure^188^, and the binding site for the E2-delivered 'donor' ubiquitin matches an additional ubiquitin-binding interface mapped in Parkin^184^. Intriguingly, in the HOIP structure, an additional, unmodified, ubiquitin occupies an identical binding site as Ser65-phosphoUb in Parkin (Figure 8C). While it is unclear whether HOIP undergoes similar conformational changes upon binding to this putative 'activator ubiquitin', the additional ubiquitin suggests that other RBR E3 ligases may be regulated by an 'activator ubiquitin', which in case of Parkin is an activator Ser65-phosphoUb. The RBR E3 ligase HHARI binds to and is activated by NEDD8^192,193^, and it is tempting to speculate that NEDD8 serves a similar role as that of Ser65-phosphoUb in Parkin activation.

Finally, Ser65-phosphoUb-mediated release of the Ubl domain of Parkin not only unblocks the E2 binding site, but also enables its phosphorylation by PINK1, adding some much needed insight into the sequence of events leading to Parkin activation^188,189,190,191^ (Figure 8C-8D). Parkin phosphorylation stabilizes it in its active form^91,188^, and we believe that the phosphorylated Ubl re-binds elsewhere on the Parkin core, covering hydrophobic surfaces exposed by the required conformational changes^188^ (Figure 8D).

## Recognizing (phospho)ubiquitinated mitochondria

In addition to recruiting and activating Parkin to significantly increase ubiquitination of mitochondria, Ser65-phosphoUb-containing chains might be the signal that distinguishes mitophagy from other forms of autophagy, as mitophagy depends on PINK1^176^. A common characteristic of autophagy receptors is their ability to bind both ubiquitin as well as Ubl modifiers of the Atg8-family that mark autophagosomal membranes^194^.

Six autophagy receptors have been annotated, namely p62/SQSTM1, NBR1, NDP52, TAX1BP1, OPTN and TOLLIP^194^. Several studies have investigated which of these proteins are recruited to mitochondria in a PINK1- and Parkin-dependent manner^176,177,195^. The studies agree on important roles for NDP52^176,177^ and OPTN^176,177,195^ (Figure 6E). Mitophagy is strongly impaired when OPTN and NDP52 are deleted from cells, and lost completely when TAX1BP1 is also deleted in a triple knockout^176^. A role for p62/SQSTM1 in mitophagy is more controversial. While the protein is recruited to damaged mitochondria^111,176,177,196,197^, it does not appear to initiate mitophagy but instead promotes mitochondrial clustering^196,197^. Autophagy receptors are expressed in a cell-type specific fashion, which may explain some of the discrepancies and observed redundancies, such as the recently reported role of p62/SQSTM1 in macrophage mitophagy after LPS stimulation^198^.

Recruitment of autophagy adaptors to mitochondria depends on their ubiquitin binding properties and also on PINK1^176,177^, suggesting that recognition of Ser65-phosphoUb is important. A preference for Ser65-phosphoUb binding could so far not be established *in vitro*, and additional factors or modifications on the receptors may be required^176,177^. Of particular importance is the protein kinase TBK1, which binds and phosphorylates many receptors, and modulates their adaptor functions^176,177,194,199^. However, how mitophagy adaptors work on mitochondria remains unclear, and a *bona-fide* Ser65-phosphoUb receptor appears to still be missing. An even bigger question is whether Ser65-phosphoUb is involved in alternative forms of autophagy, such as xenophagy (the removal of pathogens from the cytosol^200^), which uses the identical receptors NDP52 and OPTN. As mentioned above, Parkin has been linked to bacterial restriction pathways^98^, but it is unclear how it can be activated in other contexts.

## Antagonizing (or spatially restricting) mitophagy

Bulk mitophagy as induced by depolarizing reagents is far from being physiological. In a real-life scenario, a mitochondrion, or a part of the continuously fusing and dividing mitochondrial network, is damaged, and this invokes mitophagy by the mechanisms described above, yet in a spatially restricted way. How an area of mitochondria can be tagged for disposal is unclear, but this could involve negative regulators of mitophagy. In principle, the mitophagy initiation signal, i.e., polyubiquitin comprising Ser65-phosphoUb molecules, could be antagonized by DUBs, or by ubiquitin phosphatases. While the identity of the latter is unknown, several DUBs have been proposed to directly regulate Parkin-mediated mitophagy initiation, including USP30^93,95,96^, USP35^201^, USP15^202^, USP8^92^ and Ataxin-3^203^ (Figure 6). The mechanisms of these enzymes are different: USP8 and Ataxin-3 were proposed to remove polyubiquitin from Parkin itself to stabilize the enzyme, and knockdown of USP8 prevents Parkin recruitment to mitochondria^92,203^. USP30 and USP15 were suggested to target Parkin substrates on mitochondria. USP8, USP15, and Ataxin-3 have many differing roles in cells^14^, and are not dedicated to mitophagy/mitochondrial maintenance. The version of USP35 that was reported to antagonize mitophagy ('short-USP35') appears to lack a functional catalytic domain. Short-USP35 contains a mitochondrial targeting sequence (MTS) but is rapidly lost from mitochondria upon depolarization, and does not affect Parkin recruitment^201^. USP30 also contains an MTS and permanently resides on mitochondria to restrict mitophagy^93,95,96^. USP30 appears to be important in mitochondrial homeostasis as it actively removes ubiquitin from substrates such as TOM20 on healthy mitochondria (Figure 6A). This suggests that USP30 activity is switched off in mitophagy, which could occur via Parkin-mediated USP30 ubiquitination^95^ (Figure 6D).

Interestingly, and consistent with their functions, Parkin and USP30 have a matching chain linkage preference, and make or cleave Lys6-linked chains preferentially^44,93^ (see above). How Ser65-phosphoUb-containing chains are cleaved is less clear, since many DUBs are significantly less active against polyubiquitin containing exclusively Ser65-phosphoUb^44,45^. Finally, USP30 has also been shown to act as a negative regulator of Parkin-induced apoptotic cell death^96^, suggesting that it serves as a critical modulator of not only mitophagy, but also in the maintenance of overall cellular health.

## Conclusions, lessons and future outlook

Our understanding of the ubiquitin code has become much more sophisticated. Initially regarded as a defined means to degrade proteins, ubiquitination is now considered the most versatile protein modification system, impacting virtually any realm of life sciences. The discovery of many linkage-specific proteins and enzymes has alerted researchers that particular linkages must play distinct roles. The appreciation of the intricacies in the ubiquitin code has generated new methods and enabled routine checking of which linkage types are involved in particular systems. This led to new discoveries linking unstudied atypical chain types to new cellular processes, and has remarkably also started entirely new research areas, e.g., on ubiquitin phosphorylation. While information on several ubiquitin modifications is still scarce, the example of Met1-linked chains, or Ser65-phosphoUb signaling in mitophagy shows how combined efforts from many laboratories can lead to real progress in important research fields.

Chemical biology has enabled access to all ubiquitin linkage types as well as to new ubiquitin modifications, and will continue to provide essential tools to study the system as a whole. Yet, to study atypical chains or new modifications, the key was the identification of the enzymatic machineries that generate the modification, again, exemplified by LUBAC and PINK1. The recent rush in identifying new E3 ligases for atypical chains will likely prove essential to associate roles to these unstudied signals. It will also be essential to identify the enzymes regulating ubiquitin phosphorylation and acetylation for real progress to be made in these areas.

Indeed, most of the new insights into linkage-specific 'writers', 'readers' and 'erasers' of the ubiquitin code, originate in bottom-up biochemical approaches, followed by mechanistic studies by structural biology and biophysical techniques. The ubiquitin field is rich in mechanism, but somewhat lags behind in physiology. It is important that the biochemical facts and molecular details are incorporated in the physiological studies of the respective proteins; this is sometimes still missing from the current literature.

This review deals predominantly with the individual modifications of the ubiquitin molecule, and together, the distinctly modified ubiquitin moieties could be seen as the 'words' in the ubiquitin code. A further, even bigger and more challenging frontier awaits in studying the 'grammar' of the ubiquitin code. What is the architecture of polyubiquitin chains? How long can ubiquitin chains become in cells? How can we start to study mixed-linkage and branched chains, and do these polymers function differently? Are there underlying rules, akin to the hierarchy in modifications in the histone code, that exclude or enable combinations of modifications? How many combinations of modifications do we need to consider, and which ones can be detected in cells? Answering these questions requires the development of new technologies and methods, but addressing them will provide fundamental insights into ubiquitin and cellular biology.

While the ubiquitin system appears to be of mind-boggling complexity, the progress in the last decade is astounding and encourages us that the ubiquitin code can be cracked in the next few decades. We would hope that eventually, distinct modifications can be rapidly distinguished, architecture and interplay between modifications can be assessed, and downstream outcomes of the modifications can be predicted with confidence.

## Competing Financial Interests

DK is part of the DUB Alliance that includes Cancer Research Technology and FORMA Therapeutics, and is a consultant for FORMA Therapeutics.
